# Mechanistic Computational Models of Epithelial Cell Transporters-the Adorned Heroes of Pharmacokinetics

**DOI:** 10.3389/fphar.2021.780620

**Published:** 2021-11-04

**Authors:** Jasia King, Stefan Giselbrecht, Roman Truckenmüller, Aurélie Carlier

**Affiliations:** ^1^ Department of Cell Biology-Inspired Tissue Engineering, MERLN Institute for Technology-Inspired Regenerative Medicine, Maastricht University, Maastricht, Netherlands; ^2^ Department of Instructive Biomaterials Engineering, MERLN Institute for Technology-Inspired Regenerative Medicine, Maastricht University, Maastricht, Netherlands

**Keywords:** transporter, computational mechanistic models, epithelial membrane, lumped parameter, pharmacokinetics

## Abstract

Epithelial membrane transporter kinetics portray an irrefutable role in solute transport in and out of cells. Mechanistic models are used to investigate the transport of solutes at the organ, tissue, cell or membrane scale. Here, we review the recent advancements in using computational models to investigate epithelial transport kinetics on the cell membrane. Various methods have been employed to develop transport phenomena models of solute flux across the epithelial cell membrane. Interestingly, we noted that many models used lumped parameters, such as the Michaelis-Menten kinetics, to simplify the transporter-mediated reaction term. Unfortunately, this assumption neglects transporter numbers or the fact that transport across the membrane may be affected by external cues. In contrast, more recent mechanistic transporter kinetics models account for the transporter number. By creating models closer to reality researchers can investigate the downstream effects of physical or chemical disturbances on the system. Evidently, there is a need to increase the complexity of mechanistic models investigating the solute flux across a membrane to gain more knowledge of transporter-solute interactions by assigning individual parameter values to the transporter kinetics and capturing their dependence on each other. This change results in better pharmacokinetic predictions in larger scale platforms. More reliable and efficient model predictions can be made by creating mechanistic computational models coupled with dedicated *in vitro* experiments. It is also vital to foster collaborative efforts among transporter kinetics researchers in the modeling, material science and biological fields.

## Introduction

Epithelial cells form a selective barrier, permitting the controlled transport of solutes across the cell membrane via various transport mechanisms, including solute transport through transporter proteins. These transporters play a pivotal role in the pharmacokinetics of solutes. Transporters are widely studied from the perspective of toxin removal (Howe et al., 2009; Wilmer et al., 2009; Jansen et al., 2016), understanding drug development and solute interactions (Giacomini et al., 2010; Mihaila et al., 2020), *in vitro*/organ-on-chip development (Bens and Vandewalle, 2008; Chevtchik et al., 2016; Nieskens and Wilmer, 2016), solute transport based on sex differences (Veiras et al., 2017; Prasad, 2019; Hu et al., 2021), or disease models (Hediger et al., 2013; Layton, 2019; Jetter and Kullak-Ublick, 2020). However, despite extensive *in vitro* functionality studies over the past decade, only 24 out of the 400 genetically identified transporters have been characterized by mechanism, chemical configuration, and physical structure, and classified into two main categories, the ATP-Binding Cassette (ABC) and the SoLute Carrier (SLC) superfamily (Giacomini et al., 2010).

The ABC transporters are a superfamily of transmembrane proteins primarily located in the apical cell membrane and actively export metabolized solutes from the cell’s cytosol to the extracellular fluid by primary active transport (direct energy use of ATP hydrolysis) (Juliano and Ling, 1976; Schinkel et al., 1997; Doyle et al., 1998; Hipfner et al., 1999; Borst et al., 2000; Maliepaard et al., 2001). The SLC superfamily of transporters covers a more functionally and structurally diverse group located on both the apical and basolateral cell membranes. SLC transporters are responsible for the uptake and efflux of organic and inorganic solutes via facilitated transport or secondary active transport (Hediger et al., 2004; Giacomini et al., 2010). In addition, SLC transporters can transport solutes with different protein configurations, resulting in *polyspecific* transporters (Volk, 2014).

Every organ is characterized by particular modes of solute transport, which depend heavily on various combinations of SLC and ABC transporters on their epithelial cell membranes (Giacomini et al., 2010; Liu and Liu, 2013; Jansen et al., 2016; Morris et al., 2017; Layton, 2019; Jetter and Kullak-Ublick, 2020). In the specific case of renal epithelial transport, the proximal tubule clearance of anionic solutes, such as indoxyl sulfate or hippuric acid, depends on the uptake by a subset of SLC transporters (i.e., organic anionic transporters) on the basolateral membrane (Jha et al., 2013; Wang and Sweet, 2013; Volk, 2014; Nigam, 2018). After being taken up by the proximal tubule cells, these anionic solutes are then pumped out of the cell into the (pro-) urine by the efflux pumps belonging to the ABC superfamily (Giacomini et al., 2010; Jansen et al., 2016). Importantly, the proximal tubule clears more than just organic anionic solutes with these membrane transporters (Prasad et al., 2016; Hu et al., 2020). There are over 130 studied uremic solutes (according to the EUTox Database) that can interact with each other and compete for the same transporter. In this scenario, high-throughput *in vitro* screening becomes intractable in understanding the interactions and potential combinatorial effects of the essential transporter-solute functions. Moreover, the organ-dependent transporters are very difficult to study *in vitro* since many cells dedifferentiate and lose transporter expression once isolated from the body. Due to these complexities, many questions remain unanswered: Is the membrane transporter number or activity dependent on solute dynamics, disease state, or sex specificity? How can we distinguish the influence of the number of transporters from their activity? Additionally, what mechanisms are involved in transporter-mediated inter-organ communication or remote sensing to maintain homeostasis? Furthermore, how do we answer such questions without suitable *in vitro* models that sustain long-term transporter expression in culture? Interestingly, mechanistic computational models enable researchers to replicate the epithelial transport phenomena, including the solute-transporter interactions, providing avenues to explore multiple currently impossible scenarios to measure *in vitro* or *in vivo*.

Here, we aim to provide the scientific community with a mini-review that can be used to outline computational modeling of epithelial transport using different methods at the cell scale. We also aim to provide a starting point for creating new mechanistic computational models, building on existing models, and summarizing the open questions in the field. We do not discuss models of transporter crystalline structures (Chang et al., 2006), transporter protein configuration (Marger and Saier, 1993; Saier et al., 2006; Arinaminpathy et al., 2009; Almeida et al., 2017), or data-driven techniques (Seve et al., 2004), based on, for example, genomics data (Karlgren et al., 2012). For these topics, we refer the reader to the cited literature.

## Understanding Epithelial Membrane Transport Phenomena: Current Models, Assumptions, and Limitations

### Mathematical Background

We can improve our fundamental understanding of epithelial transport mechanisms and their underlying mechanisms using transport phenomena equations. Furthermore, mechanistic models generally deal with multiple variables and (non-linear) interactions, and as such, are ideal for investigating membrane transporter function and solute interactions. The following paragraph provides an overview of the various transport phenomena formulations used to model the transport across a membrane (i.e., the diffusive or reactive flux as a boundary condition).

Mechanistic transport phenomena models utilize reaction-diffusion-advection equations in the model’s spatial compartment (Eq. 1). In spatial models, transport phenomena-related processes can be diffusive [Fickian diffusion $D_{n}\left(\nabla^{2}C_{n}\right)$ ], convective [carrying of solute is flow-dependent- $v_{n}\cdot \left(\nabla C_{n}\right)$ ], and reactive (solute transformation favors chemical equilibrium- $R_{n}$ ) (Kim, 2020). The boundary conditions are prescribed at the interface between two spatial compartments and describe how the interface connects the transport of the simulated solutes at either side of the interface. Boundary conditions are typically applied as a molar flux in biological applications, as shown in Eq. 2, in which flux continuity is assumed between the diffusive flux ( $D_{n}\nabla C_{n}$ ) and reactive flux ( $J_{M}$ ). *Flux* is an important term in transport phenomena models and is defined as a vector (magnitude and direction) quantity of a substance’s flow over a unit area per unit time located at a compartment’s boundary

Transport Phenomena Equation:

$$\;\frac{\partial C_{n}}{\partial t}=-\underset{\mathrm{Convective Term}}{\underset{︸}{v_{n}\cdot \left(\nabla C_{n}\right)}}+\underset{\mathrm{Diffusive Term}}{\underset{︸}{D_{n}\left(\nabla^{2}C_{n}\right)}}+\underset{\mathrm{Reacting Term}}{\underset{︸}{R_{n}}}$$ 1

General Boundary Equation:

$$D_{n}\nabla C_{n}=J_{M}$$ 2

In the general equation, the solute is the subscript n, C_n_ [M] denotes the concentration of the species, *D*_*n*_ [m^2^.s^−1^] is the diffusivity, and $J_{M}$ [molecules.m^−2^.s^−1^] is the flux term, representing the source or sink contributions of the species. The reaction term ( $J_{M})$ models whether there is a species sink (J_M_ < 0) or source (J_M_ > 0).

**FIGURE 1:**
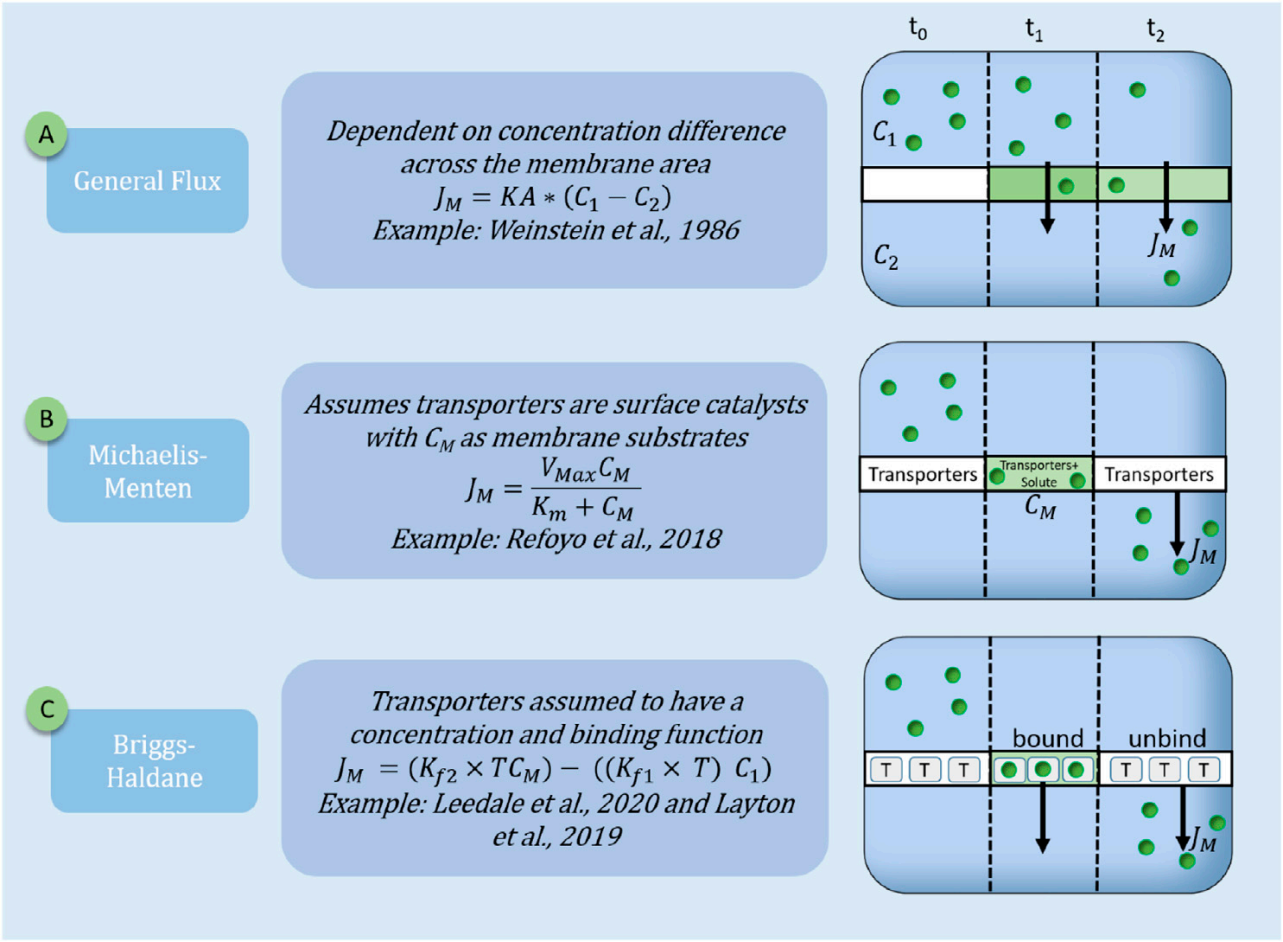
Schematic representation of three transporter models. Transporter models are represented by the flux boundary conditions of the transporter membrane over three time-steps (t_0_ = initial time point; t_1_ = intermediate and t_2_ = steady-state). J_M_ is the resulting membrane flux (molecules.cm^−2^.s^−1^). The three standard equations are: **(A)** General Flux: with KA as the mass transfer area coefficient, C_1_ and C_2_ are the concentrations of the solute in the compartments; **(B)** Michaelis-Menten: with C_M_ is the solute concentration in the membrane, K_m_ and V_max_ are the Michaelis-Menten constants; **(C)** Briggs-Haldane (mass action): with T is transporter concentration; TC_M_ is the solute bound transporter; $K_{f1}$ and $K_{f2}$ is the on and off binding rate of the transporters with the solute.

For a spatial model, the flux term of the general equation (Eq. 2), captures the membrane transporter interactions with the simulated solutes. In a non-spatial setting, boundary conditions are not required and the membrane transporter solute interactions are captured with the Reacting term (Eq. 1). The reaction term ( $R_{n})$ or flux term ( $J_{M})$ , depending on a non-spatial or spatial formalism respectively, can take various kinetic forms (as depicted in Figure 1):

Flux continuity: $D_{2}\nabla C_{2}$

  • Where the interface between the compartments is considered to be open. Such that, the concentration of the solute leaving compartment 1 ( $C_{1}$ ) is equal to the concentration of the solute entering compartment 2 ( $C_{2}$ ).
General Flux: $\mathrm{KA*}\left(C_{1}-C_{2}\right)$ .
  • Describes the bulk movement of solutes across a membrane using a variation of Fickian diffusion. The driving force is the concentration difference of the solute across the membrane, and it relates to the flux of the membrane by the proportionality constant called the area mass transfer coefficient (KA).
Michaelis-Menten: $\frac{V_{\mathrm{Max}}*C_{M}}{K_{m}+C_{M}}$

  • The Michaelis-Menten kinetic formulation is often proposed for membrane binding kinetics for the transport across the epithelial cell membrane. $V_{Max}$ is the maximum reaction rate at saturation, while $K_{m}$ is considered as half of the maximum reaction rate (Viaenea et al., 2013).
Briggs-Haldane or law of mass action: $\left(K_{f2}\times TC_{M}\right)-\;\left(\left(K_{f1}\times T\right)\;C_{1}\right)$ .
  • Where T is the transporter density, $TC_{M}$ is the concentration of solute bound to the transporter. Describes the forward ( $K_{f1}$ ) and reverse ( $K_{f2}$ ) binding kinetics of the biological phenomenon of the membrane transporter bound species ( $TC_{M}$ ) dissociating to the compartment species ( $C_{1}$ ). $K_{f1}$ and $K_{f2}$ are related to the equilibrium constant as $K_{\mathrm{eq}}=\;\frac{\left[\mathrm{Products}\right]}{\left[\mathrm{Reactants}\right]}=\;\frac{K_{f1}}{K_{f2}}$ . The reaction rate can be temperature-dependent, whereby the reaction rate can be zeroth-order (independent of reacting concentrations), first-order (linearly dependent on one reacting solute concentration), or second-order (dependent on the square of the reacting solute concentration) (House, 2007).

### Assuming Transport Across a Membrane is Independent of Transporter Concentration

Current mechanistic models of the ABC and SLC superfamilies are limited to lumped parameters or reduced models to describe the transport function and their influences on solute transport (Weinstein, 1986; Refoyo et al., 2018; Ito et al., 2020). For example, Weinstein (1986) developed a model to investigate the transport of solutes in the proximal tubule (PT) and distal convoluted tubule (DCT). Here, Weinstein modeled the reabsorption of Cl^−^, HCO3^−^, HPO4^2−^, H2PO4^−1^, glucose, urea, Na^+^, and K^+^ across the epithelial membrane as a general flux dependent on solute concentration, flow rate, epithelial area, and membrane electrical conductance (see Figure 1A). The general solute flux (JB) was one of the many fluxes included, modeling the entire rat proximal tubule reabsorption. Using this model, Weinstein predicted K^+^ by a diffusive and convective flux, with a lag in the K^+^ convective flux in the early segment of the tubule, which corresponds to the previous experimental reports in rats (Weinstein, 1986).

Another lumped model was published by Refoyo et al. (2018), where they approximated indoxyl sulfate transport by the basolateral SLC22A6 transporter with Michaelis-Menten kinetics (see Figure 1B) to represent the transport mechanism in a perfused bioartificial kidney. They used a parametric study to identify particular model parameters such as the active transport kinetics through the cell monolayer as this information was not experimentally available. They concluded that the concentration of the SLC22A6 transporters (represented by the Michaelis-Menten kinetic parameters) was the most influential parameter on indoxyl sulfate clearance.

Michaelis-Menten kinetics is often applied to transporters and can also be seen in a model developed by Howe et al. (2009). Here, they developed a carboxydichlorofluroscein (CDF) transport model by the ABCC2 transporter in a hepatocyte. They used a mixture of parameter values available in the literature and experimentally determined through fitting their model to the experimental data. Their model development was similar to Refoyo et al. as they use Michaelis-Menten kinetics to lump the ABCC2 function and expression parameter values. Their simulations resulted in ABCC2 having the most significant influence on the flux of CDF.

### Assuming Transport Across a Membrane is Dependent on Transporter Concentration

More recent mechanistic models consider the transporter as a separate variable in the flux formulation. Layton and Layton (2019) developed a model to predict the transport of 15 solutes in the various nephron segments. They included the dependency of solute transport on solute concentration, fluid flow and transcellular, and paracellular fluxes. The model is developed with an extensive system of ordinary differential equations and algebraic expressions at steady-state. Moreover, they represented the SGLT2 transporter density as being linearly dependent on flow. As such, the number of transporters can change within the compliant tubule depending on the flow conditions. Using this model, they investigated SGLT2 and NKCC2 transporter inhibition in humans, which is impossible to measure *in vivo* due to ethical limitations. They used available parameters from rat renal physiological models to overcome this issue and made appropriate scaling-up assumptions for the human tubules’ geometry, transporter numbers, and flow rate. The model predictions were then successfully validated by comparing human urine clearance data to produce an accurate human proximal tubule mechanistic model. Finally, Layton et al. used the model to predict the effects of a diabetic treatment on the human proximal tubule through the inhibition of SLGT2 (Layton and Layton, 2019) and sex-difference effects on solute transporters (Hu et al., 2020, 2021).

Another model that displays excellent specificity in describing transport function is the research conducted by Leedale et al. (2020). In 2D and 3D spheroids, the liver transporters are modeled as microscale kinetic reactions between generic liver transporters and a generic drug. They included both passive and carrier-mediated transport as a boundary condition at the cell membrane. The carrier-mediated transport was explicitly modeled to incorporate the density of the membrane transporters, T0, and the multiscale effects of geometry on membrane transport. They investigated the spatio-temporal hepatic drug penetration dynamics by capturing varying metabolism rates in selected liver organoid zones. Their predictions can be used to inform the optimal dosages and delivery system required for *in vivo* experiments involving lipophilic drugs.

Modeling the transporters explicitly is instrumental when investigating the effects of disease on epithelial membrane transport. Afshar et al. (2019) developed and validated a mechanistic model of the glucose uptake by the SLGT1 and GLUT2 (both categorized in the SLC family) present in the small intestine. First, they were able to test the hypothesis that there is a varying combination of SLGT1 and GLUT2 expression along the epithelial membrane to capture the change in apical glucose concentration uptake kinetics above 10 mM. Next, they expanded the model to develop a diabetic model using a three-fold increase of SLGT1 and GLUT2 expression. The results suggested that GLUT2 expression has a more critical role in glucose uptake in a diabetic patient than SLGT1. Importantly, these hypotheses required that glucose flux across the epithelial membrane was modeled as a transporter expression function.

### Applications and Limitations: Use of Transporter Independent or Dependent Models

It is critical to select and develop an epithelial transport model that optimally aligns with the model scale, scope of research, and data availability. For example, when observing the overall input and output of the nephron, and not the individual transporter kinetics, as seen in Weinstein (1986) (Table 1), it is beneficial to use a generalized flux term for solute transport as it is part of the broader scope of the research question. However, in the Refoyo et al. (2018) and Howe et al. (2009) models (Table 1), they wanted to explore the importance of membrane transporters on solute clearance and thus use Michaelis-Menten kinetics, capturing the activity and amount of transporters (albeit in a lumped fashion). Michaelis-Menten kinetics is a standard kinetic formulation used to describe membrane transport in pharmacokinetics as it assumes the rate of transport increases non-linearly with the reacting solute concentration. It should be noted that the Michaelis-Menten equation was initially developed to describe enzyme kinetics and has a list of underlying assumptions that are sometimes neglected when applied to transport phenomena (Keener and Sneyd, 2009; Kim and Tyson, 2020).

**TABLE 1:** Differences in model results when using mechanistic computational models with a flux boundary condition dependent on transporter density or not by various researchers.

Type	Application	Model specifications	Example References
Transporter independent flux	Organ model development	Transport of multiple solutes across the nephron cell membrane, where the membrane flux was dependent on electrical conductance and solute concentration gradients	Weinstein, (1986)
	Device design	Improving clearance rates of indoxyl sulfate in the media perfused hollow fiber membrane of a bioartificial kidney	Refoyo et al. (2018)
	Estimate drug impact	The model was used to rapidly estimate the inhibitory value of CDF and investigate the impact ABCC2 has on the flux of CDF across the hepatocytes	Howe et al., 2008
Transporter dependent flux	Genetic differences	Transporter expression is dependent on the sex differences in species	Hu et al. (2020)
	Disease state	The model alters the expression of GLUT2 and SLGT1 to investigate their contribution to glucose transport in a diabetic patient, resulting in GLUT2 expression being most important for glucose	Afshar et al. (2019)
	Flow rate effects	Models change in transporter expression based on stimulation by flow rate in the tubules via microvilli torque	Layton et al. (2019)

Transporter-independent flux boundary conditions are used in lumped parameter models such as device design and whole organ models. Transporter-dependent flux boundary conditions are used in a model that investigates the direct effect of the epithelial membrane transporters on membrane transport.

When using Michaelis-Menten kinetics, the researcher simplifies the mechanisms affecting the transport rate and only models the overall rate while implying transporter number effects with the Michaelis-Menten parameters. More specifically, the researcher assumes that the V_Max_ term, representing the maximal transport rate, is a product of the transporter number and activity. In other words, the number of transporters and their activity is lumped together into one rate parameter. However, the transporter activity, in reality, depends on a combination of physical, and chemical parameters, such as the solute concentration on either side of the membrane, the particular transporter mechanism, and solute interactions.

This oversimplification concern is also applicable when using a general flux term. There is no distinction between the activities of the transporters versus their number mathematically. Additionally, transporter location is often neglected by assuming a uniform distribution over the entire transporting surface. Finally, as also discussed by Zamek-gliszczynski et al. (2003), the problem with oversimplifying the transport processes in whole organ modeling is that there is an assumption of attributing the overall kinetics to the kinetics of a single transporter, when in fact, multiple transport processes may co-exist.

There are limitations when explicitly modeling transporter density along the cell membrane. For example, in Leedale et al. (2020) the hepatic uptake was modeled as being dependent on trans (Layton and Layton, 2019) porter expression and binding kinetics on a transporter scale. They modeled the membrane transporter in general [T_0_, see Eq. 2.9 in Leedale et al. (2020)] but considered the flux across the hepatocyte cell membrane as a combination of active transport and passive diffusion by altering the α_n_ terms. However, the multiscale model was simplified to account for only passive diffusion across the epithelium since more compound-specific data was needed accurately to parameterize the model at all scales entirely. Similar efforts were described by Layton and Layton (2019), where they assumed a linear relationship between transporter density and fluid flow (microvilli torque) due to lack of data availability.

The models of Layton and Layton, (2019); Hu et al., 2021, Leedale et al. (2020), and Afshar et al. (2019) (summarized in Table 1), are ideal examples of the importance of explicitly modeling the transport information to investigate the downstream effects on the entire system (see also Figure 1C). Layton and Layton (2019); Hu et al., 2021 were only able to investigate the influence of blood pressure, diabetes, and sex differences on solute transporters by explicitly including a transporter density in the functions. Similarly, Leedale et al. (2020) could only illustrate a non-linear increase in intracellular drug concentration with transporter protein concentration by explicitly modeling the transporter density. Afshar et al. (2019) predicted that the GLUT2 expression was the most influential parameter altered in a diabetic state instead of the SLGT1 expression. The results suggested that further research needs to be conducted on the expression of other transporters in diabetic patients and the effect thereon on solute transport. However, these investigations will only be possible if the transporter expression is an independent variable in the system of equations. Thus, it is evident that the solute-transporter relationship is intricate, and many factors may affect transporter concentration and function. However, an integrated understanding of the solute-transporter relationship is currently missing due to the following challenges:

1) Limited access to or inability to measure *in vivo* readouts.
2) Limited data availability due to *in vitro* experimentation challenges.
3) Limited multiscale models lead to a reduction in the accuracy of transporter mechanisms due to parameter oversimplification in lumped parameters.
4) Increased complexity of competition and solute-transporter interactions, which involve multiple possible combinations.

## Perspective and Outlook on Mechanistic Models of Epithelial Membrane Transporters

As mentioned above, one of the challenges when developing mechanistic models is the lack of adequate and relevant data for calibration and validation. This is seen in Layton and Layton (2019), where the relationship between apical flow rate and SLGT2 expression is unknown and assumed to be linear. The assumption is valid with the data currently available and the scope of the model. Since the models predict spatiotemporal phenomena, time-series data are necessary for calibration. Model validation could be done by endpoint measurements, although data-rich experiments utilizing micro (bio) sensors or microfluidic sampling allow for better validation and identification of essential model extensions.

Regarding transporter models, we do not know the exact number of transporters present on the cell membrane, nor do we know the influence of various microenvironmental cues, native to the *in vivo* epithelium, on the individual transporter rate. *In vitro* transporter studies are challenging to perform due to the lack of epithelial cell lines that robustly express all the relevant epithelial transporters and their inability to maintain these transporters systems over extended culture periods. To characterize transporter activity, researcher used indirect measurements to simplify the experiments, such as fluorescently labeled solutes, to stand in for the real physiological solutes interacting with the transporters. Quantification of transporter numbers is also a challenging process. Protein identification techniques, such as western blot analyses and mass spectrometry, only quantify relative protein content (Pelkonen et al., 2017). Image analysis techniques using immunofluorescence staining require high-resolution imaging and computational resources for quantifying the transporters on the cell membrane due to the small size of these transporters. Transporters are usually measured between 60 and 80 Å and require higher resolution imaging equipment such as stimulated emission depletion (STED) microscopy (Vicidomini et al., 2018) or freeze-fracture electron microscopy (Severs, 2007).

*In situ*, tissues are in an established hierarchy from the molecular scale up to the bulk mesoscale. Moreover, all scales influence each other, i.e., the micro- or nanoscale information predicts the macroscale concentration. Therefore, it is vital to develop mechanistic models that can couple multiple scales to explore the solute interactions from the molecular (transporter-solute interaction) to the tissue/organ scale (including communication between different tissues via electrical, metabolite sensing, or hormonal cues). For instance, as described in the remote sensing and signaling hypothesis, SLC and ABC transporter networks have interconnected pathways to sense and signal environmental changes and maintain homeostasis (Wu et al., 2011; Nigam, 2018). Furthermore, these transporter networks function together and result in multi-organ failure in disease states, such as chronic kidney disease (Torres et al., 2021). Multiscale mechanistic models could give better insights and understanding to drug handling in multiple organs and disease states, resulting in multi-organ failure.

There are some exciting multiscale developments such as the FDA-approved closed-loop artificial pancreas (Kovatchev, 2018); the Virtual Liver Project (Holzhütter et al., 2012), which simulates drug dosage, metabolism, and excretion by the liver; and, on a larger scale, the Virtual Physiological Human (Kohl and Noble, 2009) or simulation patient avatars to assist with personalized medicine (Brown, 2015).

Future work should build on the transport models mentioned above and investigate the multitude of physical and chemical cues, sex differences, remote sensing, and disease effects on the transporter number and activity by making all contributions explicit in the flux term. Modeling the explicit fundamental transporter mechanisms adds flexibility to the model investigations and captures the underlying biological mechanisms more closely than simply using lumped parameters. More integrated *in silico* and *in vitro* studies can focus on the intricate mechanisms of the solute-transporter relationship by using high-throughput platforms that allow large-scale screening of multiple solutes. Expanding accurate physiological models of transporter expression to disease or multiple scales, these models will provide critical evidence for researchers on future breakthroughs.

## Conclusion

Mechanistic computational models can help unravel the complexity of interacting spatio-temporal transport processes by providing a quantitative framework for generating and exploring research hypotheses on the governing mechanisms. By coupling mechanistic models to dedicated microphysiological *in vitro* models, a system of equations can be built to study leaky barriers, reduced efficacy of the transporters, and other inconsistencies in the *in vitro* experiments. Membrane transporter availability and function contribute directly to the overall solute flux across the epithelial cell membrane and is defined by the modeler with an appropriate boundary condition. Therefore, modelers need to critically assess how they select the type of boundary conditions applied to the epithelial membrane flux models. Both transporter-dependent and -independent flux boundary conditions are valid in epithelial membrane research. On the one hand, if a detailed model investigates the influence of sex differences (Prasad, 2019; Hu et al., 2020), disease (Hediger et al., 2004, 2013), flow rate (Kohl and Noble, 2009; Holzhütter et al., 2012; Brown, 2015) drug (Howe et al., 2009; Brown, 2015), or solute (Afshar et al., 2019) concentration on transporter expression and function, including explicitly the transporter as a variable, is essential. On the other hand, if the variables do not influence the transporter expression in the model or this is not of interest for the research question at hand, a lumped model is an excellent alternative to investigate the overall system kinetics.

As reviewed here, it is apparent that the boundary conditions selected for the transporter models depend on the research questions and the desired scope the modelers wish to investigate. The selected boundary condition directly impacts the conclusions made about the epithelial transporters. The transporter function has often been modeled as a generalized flux, making it impossible to explore and understand the intricate solute-transporter relationship. With increased available accurate data, advanced mechanistic models of transporter-dependent flux can be developed to predict the transport phenomena occurring in cell monolayers, tissues, organs, and whole humans. We believe that by tapping into advances in the field of pharmacokinetics and molecular biology through a close collaboration between modelers, materials scientists, and biologists, significant insights on transporter biology can be made.
